# Supplementary material for: VCP maintains nuclear size by regulating the DNA damage-associated MDC1–p53–autophagy axis in Drosophila
Source: Nat Commun. 2021 Jul 12;12:4258. doi: 10.1038/s41467-021-24556-0 (PMC8275807; doi:10.1038/s41467-021-24556-0)
Supplement: Supplementary file 1 — Supplementary Information [file 41467_2021_24556_MOESM1_ESM.pdf]

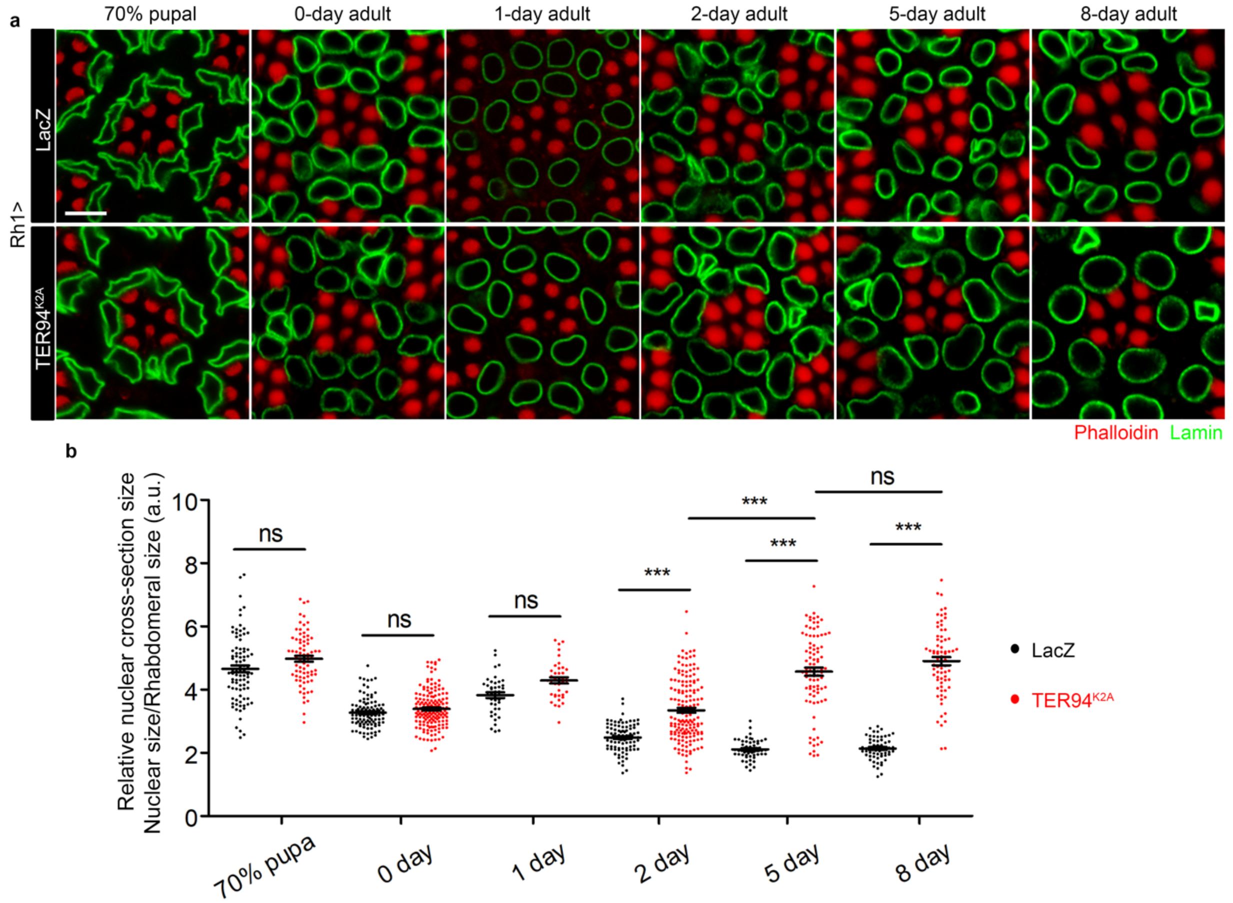

Supplementary Figure 1. Developmental changes in the morphology and size of outer photoreceptor cell nuclei. a Time-course analysis of the change in the nuclear lamina from 70% pupal to the 8-day-old adult stage. Pupal or adult *Rh1>LacZ* and *Rh1>TER94*^{K2A} eyes stained with phalloidin (red) and anti-Lamin (green) antibody. **b** Quantification of the nuclear cross-section area of R1-R6 from flies of indicated genotypes. For the comparison of the relative nuclear cross-section size, the number of independent nuclei measured are 88 (70% pupa, LacZ), 76 (70% pupa, TER94^{K2A}), 99 (0 day, LacZ), 159 (0 day, TER94^{K2A}), 42 (1 day, LacZ), 39 (1 day, TER94^{K2A}), 95 (2 day, LacZ), 162 (2day, TER94^{K2A}), 56 (5 day, LacZ), 88 (5 day, TER94^{K2A}), 62 (8 day, LacZ), and 73 (8 day, TER94^{K2A}). Values represent mean ± SE. One-way ANOVA with Bonferroni's multiple comparison test compared to LacZ at different time points. $p = 0.0751$, ns (70% pupa); $p > 0.9999$, ns (0 day); $p = 0.0701$, ns (1 day); ***$p < 1.0e{-}15$ (2day); ***$p < 1.0e{-}15$ (5 day); ***$p < 1.0e{-}15$ (8 day). Compared to 2-day TER94^{K2A}, ***$p < 1.0e{-}15$ (5 day, TER94^{K2A}). Compared to 5 day TER94^{K2A}, $p = 0.0665$, ns (8 day, TER94^{K2A}). Scale bar: 5 μm (**a**).

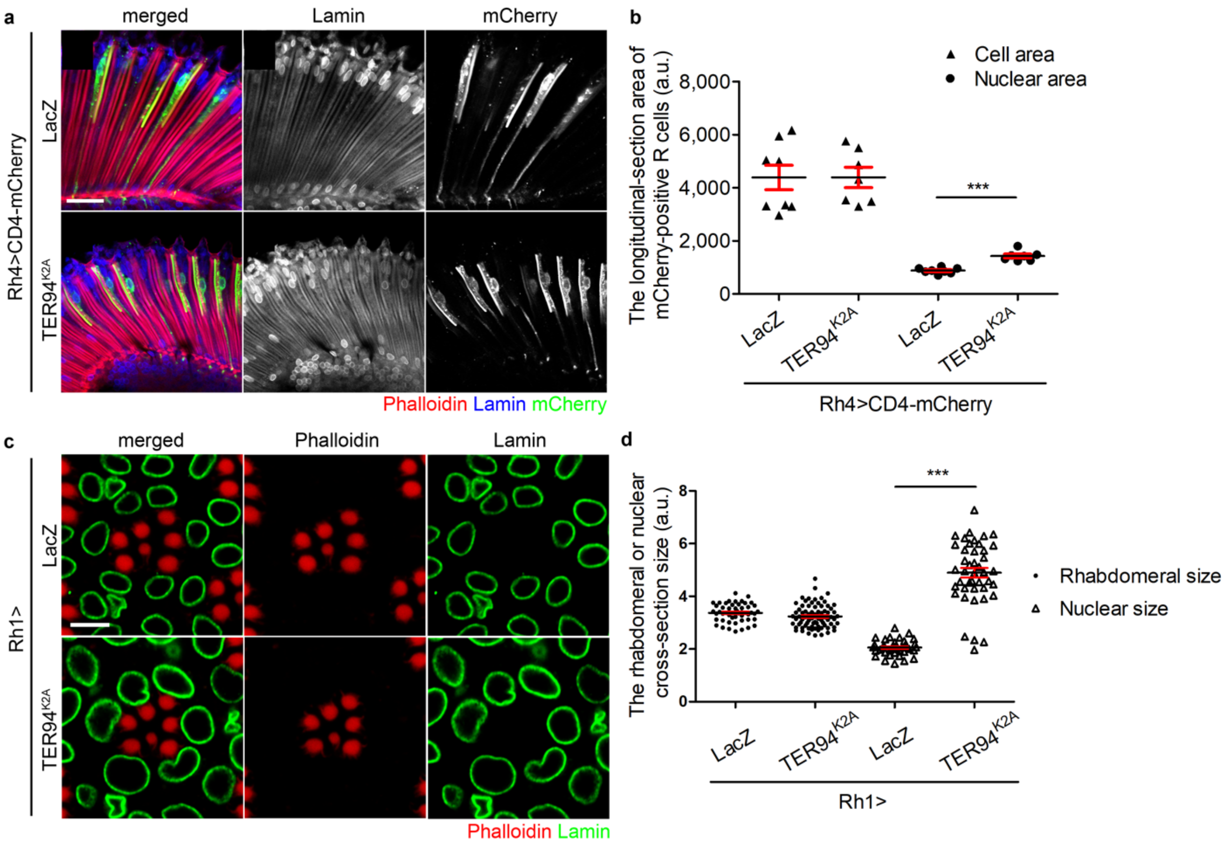

Supplementary Figure 2. Loss of TER94 function increases the nuclear size without altering the overall longitudinal cell dimensions. a Confocal images of 3-day-old *Rh1>LacZ* and *Rh1>TER94^{K2A}* adult retinas co-expressing CD4-mCherry, stained with phalloidin (red), anti-mCherry (green), and anti-Lamin (blue) antibodies to visualize the rhabdomeres, the cytoplasmic membrane, and the nuclear lamina, respectively. **b** Quantification of the cellular or nuclear longitudinal-section area of R7 cells, manually outlined according to the mCherry or anti-Lamin signals respectively as shown in (**a)** and measured by Image J. The number of independent cell area and nuclear area measured are 8 (LacZ) and 7 (TER94^{K2A}). Values shown represent mean ± SE (student's t-test, two-tailed, cell area: p = 0.9900, ns; nuclear area: ***p = 9.5e–6). **c** Confocal images of 5-day-old *Rh1>LacZ* and *Rh1>TER94^{K2A}* adult retinas stained with phalloidin (red) and anti-Lamin (green) antibody. **d** Quantification of the rhabdomeral or nuclear cross-section area of R1-R6 nuclei from flies expressing LacZ or TER94^{K2A}. The number of independent rhabdomeres measured are 50 (LacZ) and 76 (TER94^{K2A}). For the nuclei, the number are 37 (LacZ) and 43 (TER94^{K2A}). Values shown represent mean ± SE (student's t-test, two-tailed, rhabdomeral area: p = 0.0671, ns; nuclear area: ***p <1.0e–15). Scale bars: 20 μm (**a**), 5 μm (**c**).

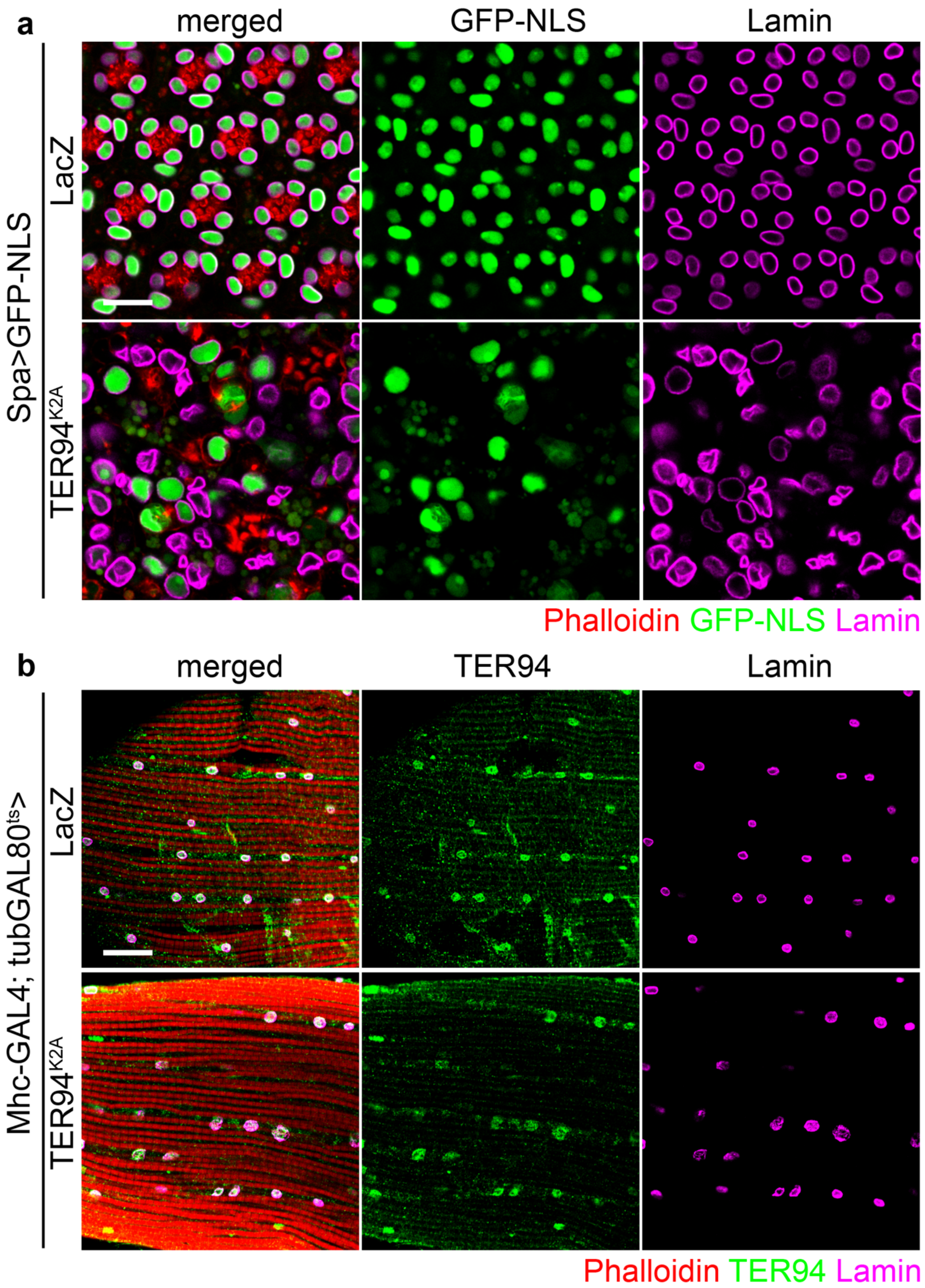

a
merged
GFP-NLS
Lamin
Spa>GFP-NLS
LacZ
TER94^{K2A}
Phalloidin GFP-NLS Lamin
b
merged
TER94
Lamin
Mhc-GAL4; tubGAL80^{ts}>
LacZ
TER94^{K2A}
Phalloidin TER94 Lamin

Supplementary Figure 3. TER94 dysfunction induces nuclear size increase in cone cells and indirect flight muscle cells. a Confocal images of late pupal retinas co-expressing GFP-NLS with LacZ (control) or $TER94^{K2A}$ in cone cells under the control of *Spa-GAL4* stained with phalloidin (red) and anti-Lamin (magenta) antibody to visualize the rhabdomeres and the nuclear envelope, respectively. GFP-NLS indicates the cone cell nuclei in the retina. **B** Adult indirect flight muscles expressing LacZ or $TER94^{K2A}$ by *Mhc-GAL4; tub-GAL80^{ts}* are stained with phalloidin (red), anti-Lamin (magenta), and anti-TER94 (green) antibodies. Flies raised at 18°C are transferred to 30°C after eclosion for 3 weeks to induce transgenes expression. Images are representative of two independent experiments. Scale bar: 10 μm (**a**), 20 μm (**b**).

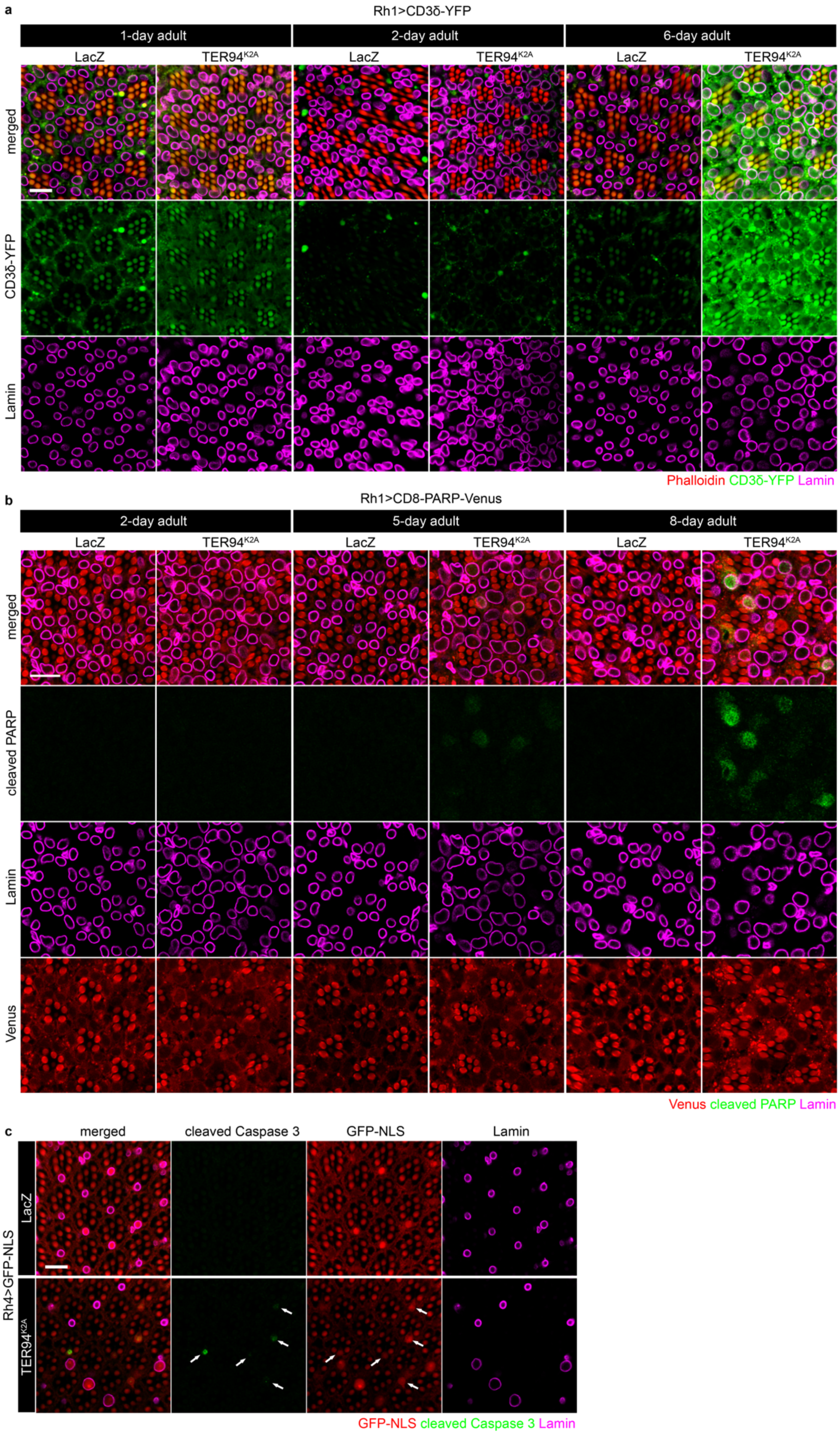

a
Rh1>CD3δ-YFP
1-day adult
2-day adult
6-day adult
LacZ
TER94K2A
merged
CD3δ-YFP
Lamin
Phalloidin CD3δ-YFP Lamin
b
Rh1>CD8-PARP-Venus
2-day adult
5-day adult
8-day adult
cleaved PARP
Venus
Venus cleaved PARP Lamin
c
merged
cleaved Caspase 3
GFP-NLS
Lamin
Rh4>GFP-NLS
GFP-NLS cleaved Caspase 3 Lamin

Supplementary Figure 4. TER94 dysfunction-caused nuclear expansion is not linked to a failure in ERAD or apoptosis activation. a Time-course analysis of the change in the nuclear size and the signal of ERAD reporter, CD3δ-YFP, from 1-day-old to 6-day-old adult stage. *Rh1>LacZ* and *Rh1>TER94^{K2A}* adult eyes co-expressing CD3δ-YFP are stained with phalloidin (red) and anti-Lamin (magenta) antibody. **b** Time-course analysis of the change in the nuclear size and the signal of apoptotic reporter, cleaved PARP, from 2-day-old to 8-day-old adult stage. *Rh1>LacZ* and *Rh1>TER94^{K2A}* adult eyes co-expressing CD8-PARP-Venus are stained with anti-cleaved PARP (green) and anti-Lamin (magenta) antibodies. The signals of Venus and anti-cleaved PARP are used to visualize the expression of the probe and caspase activation, respectively. **c** 5-day-old adult eyes co-expressing GFP-NLS with LacZ or TER94^{K2A} by *Rh4-GAL4* are stained with anti-cleaved caspase 3 (green) and anti-Lamin (magenta) antibodies. GFP-NLS marks the R7 cells with transgenes expression. The white arrows point out the cleaved caspase 3 staining in a small population of GFP-NLS-positive R7 cells. Images are representative of two independent experiments. Scale bar: 10 μm (**a**-**c**).

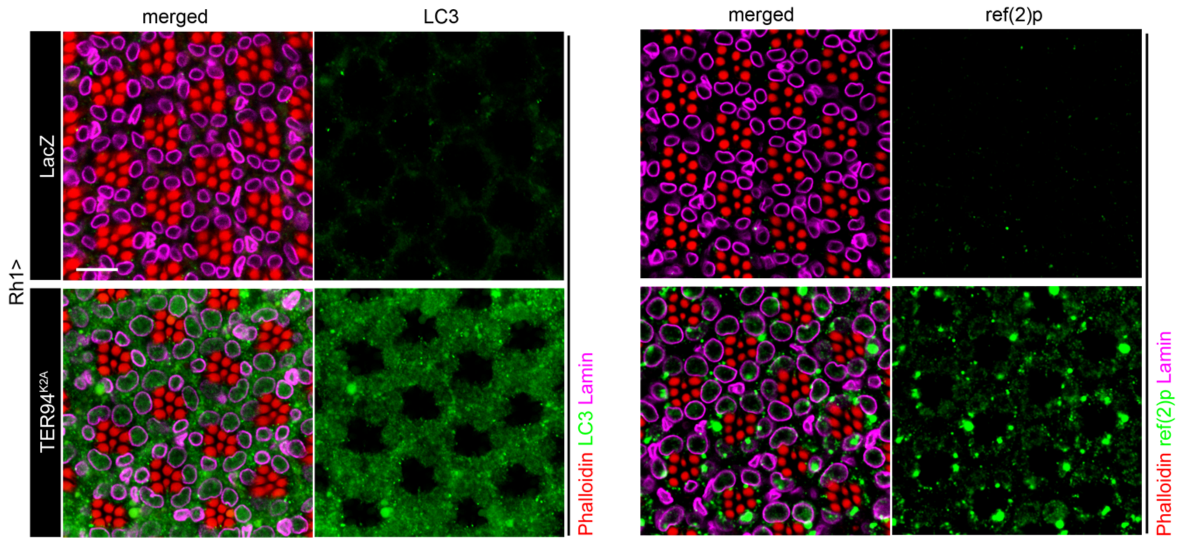

Supplementary Figure 5. LC3 and ref(2)p accumulate in TER94 dysfunction cells. 5-day-old *Rh1>LacZ* and *Rh1>TER94*^{K2A} adult retinas stained with phalloidin (red), anti-Lamin (magenta), and anti-LC3 (left, green) or anti-ref(2)p (right, green) antibodies. Images are representative of two independent experiments. Scale bar: 10 µm.

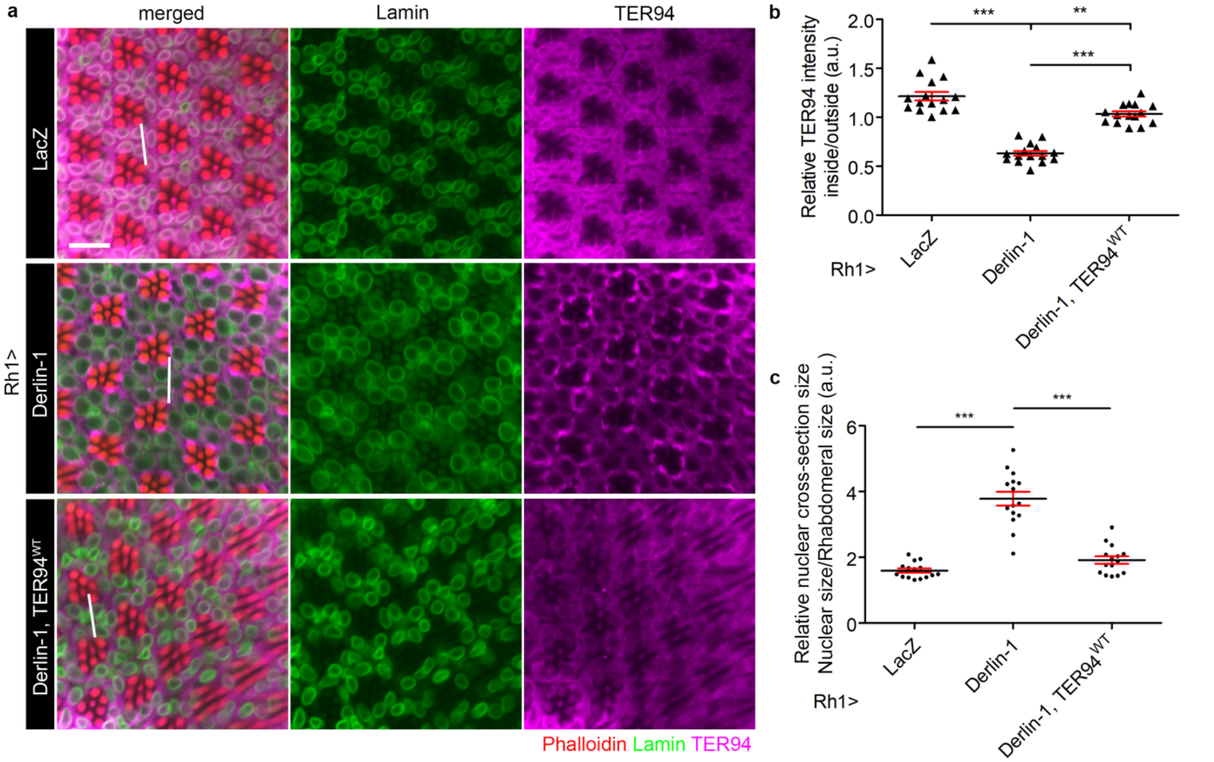

Supplementary Figure 6. Overexpressing TER94^{WT} suppresses Derlin-1-induced nuclear expansion phenotype. a Adult eyes expressing LacZ (control), Derlin-1, or co-expressing Derlin-1 with TER94^{WT} by *Rh1-GAL4* are stained with phalloidin (red), anti-Lamin (green) and anti-TER94 (magenta) antibodies. **b** Quantification of the relative anti-TER94 intensity inside versus outside the nucleus in tissues of indicated genotypes. The intensity of anti-Lamin and anti-TER94 signals along the lines is measured by Image J and the peaks of anti-Lamin signals are used to define the borders of the nuclei. 15 random analyzing regions from 3 independent eyes are measured in each group. Values represent mean ± SE. One-way ANOVA with Bonferroni's multiple comparison test compared to LacZ. ***p < 1.0e–15 (Derlin-1); **p = 0.001 (Derlin-1, TER94^{WT}). Compared to Derlin-1, ***p < 1.0e–15 (Derlin-1, TER94^{WT}). **c** Quantification of the nuclear cross-section area of R1-R6 from flies of indicated genotypes. 15 nuclei from 3 independent eyes are measured in each group. Values represent mean ± SE. One-way ANOVA with Bonferroni's multiple comparison test compared to LacZ. ***p < 1.0e–15 (Derlin-1). Compared to Derlin-1, ***p < 1.0e–15 (Derlin-1, TER94^{WT}). Scale bar: 10 μm (**a**).

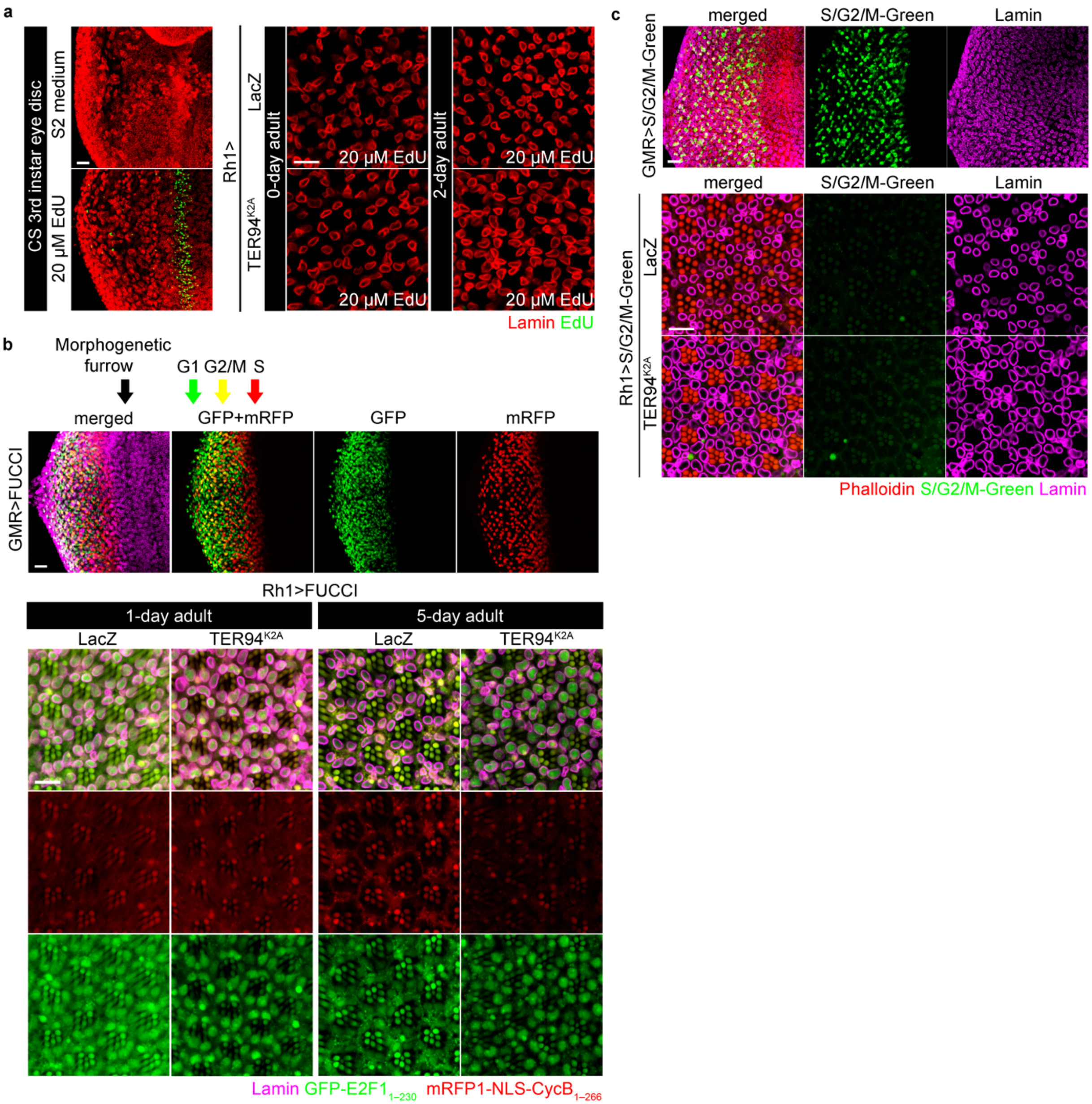

Supplementary Figure 7. The nuclear expansion induced by TER94 dysfunction is not coupled to cell-cycle re-entry. a EdU cell proliferation assay. Images of 3rd instar Canton S (CS) larval eye discs (left panel) containing proliferating cells incubated with S2 medium (negative control) or 20 μM EdU (positive control) for 1.5 hrs prior to fixation. The right panel shows adult retinas expressing LacZ or TER94^{K2A} by *Rh1-GAL4* subjected to the EdU incorporation assay before (0-day, freshly-eclosed) and after (2-day) the nuclear expansion occurs. The anti-Lamin (red) antibody is used to mark the nuclear envelope and the Alexa Fluor® 488 azide (green) is used to detect the incorporated EdU. **b** Examination of the cell-cycle status with Fly-FUCCI, a fluorescent cell-cycle indicator composed of GFP-E2F1$_{1–230}$ and mRFP1-NLS-CycB$_{1–266}$. This technique differentially labels cells in the G1 (green; degradation of mRFP-tagged CycB), S (red; degradation of GFP-tagged E2F), and G2/M (yellow) phases. *GMR>Fly-FUCCI* larval eye discs (upper panel) show the cells behind the morphogenetic furrow

in the S, G2/M, and G1 phases are labeled by green, yellow, and red fluorescence, respectively, as the corresponding arrows indicated. *Rh1>LacZ* or *Rh1>TER94*^{K2A} adult retinas (lower panel) co-expressing Fly-FUCCI before (1-day) and after (5-day) the nuclear expansion are stained with anti-Lamin (magenta) antibody to visualize the nuclear envelope. **c** Confocal micrographs of larval and adult eyes expressing S/G2/M-Green, a cell-cycle indicator labeling cells in the S, G2, or M phases of cell cycle with green fluorescence. Larval eye discs (upper panel) expressing S/G2/M-Green by *GMR-GAL4* are included as a positive control. Adult retinas (lower panel) co-expressing S/G2/M-Green with LacZ or TER94^{K2A} under the control of *Rh1-GAL4* are stained with phalloidin (red) and anti-Lamin antibody (magenta). Images are representative of 2 (S2 medium) and 7 (EdU) eye discs, 3 (LacZ) and 4 (TER94^{K2A}) 0-day adult eyes, and 3 (both LacZ and TER94^{K2A}) 2-day adult eyes in **a**. Images in **b** are representative of three independent experiments. Images in **c** are representative of 4 (eye discs) and 3 (adult eyes) independent experiments. Scale bar: 20 μm (**a**-**c** eye discs), 10 μm (**a**-**c** adult retinas).

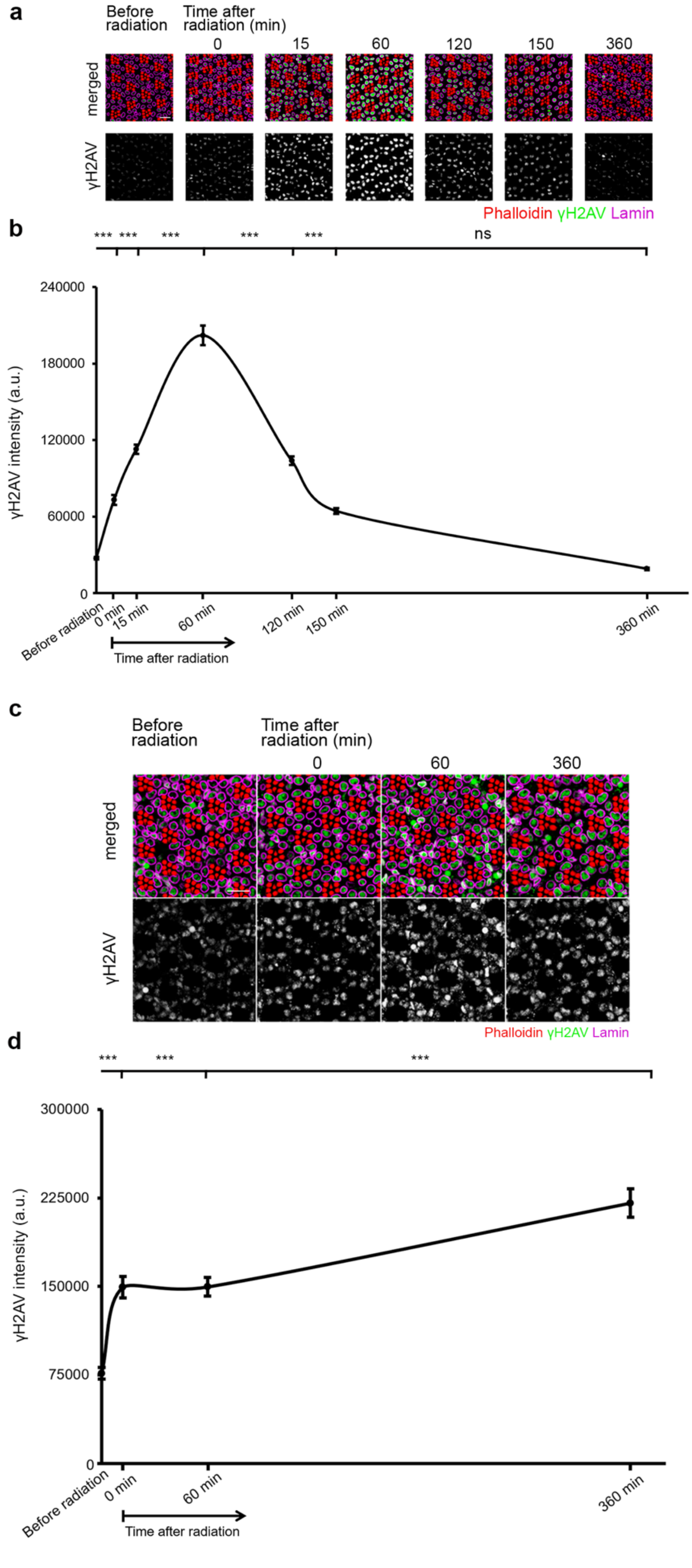
a
Before radiation
Time after radiation (min)
0
15
60
120
150
360
merged
γH2AV
Phalloidin γH2AV Lamin
b
*** *** *** *** ***
ns
240000
180000
120000
60000
0
γH2AV intensity (a.u.)
Before radiation
0 min
15 min
60 min
120 min
150 min
360 min
Time after radiation
c
Before radiation
Time after radiation (min)
0
60
360
merged
γH2AV
Phalloidin γH2AV Lamin
d
*** *** ***
300000
225000
150000
75000
0
γH2AV intensity (a.u.)
Before radiation
0 min
60 min
360 min
Time after radiation

Supplementary Figure 8. The dynamics of R cells' DNA damage repair after exposure to 1 Gy γ-radiation. a, c Confocal images of 5-day-old wild type (**a**) and 2-day-old *Rh1>TER94*^{K2A} (**c**) adult flies before and after the exposure of total 1 Gy ionizing radiation dose at the indicated recovery timepoints. The retinas are stained with phalloidin (red), anti-Lamin (magenta), and anti-γH2AV (green) antibodies. The anti-γH2AV signals are shown separately and converted to black and white for comparison. Scale bars: 10 μm. **b, d** Quantification of the anti-γH2AV signal intensity from flies of indicated timepoints. (**b**) The number of independent cells measured are 235 (before radiation), 169 (0 min), 176 (15 min), 192 (60 min), 170 (120 min), 221 (150 min), and 170 (360 min). Values shown represent mean ± SE. One-way ANOVA with Bonferroni's multiple comparison test compared to "before radiation" (0 min, ***p < 1.0e–15; 15 min, ***p < 1.0e–15; 60 min, ***p < 1.0e–15; 120 min, ***p < 1.0e–15; 150 min, ***p < 1.0e–15; 360 min, p = 0.4681, ns, not significant). (**d**) The number of independent cells measured are 64 (before radiation), 66 (0 min), 64 (60 min), and 57 (360 min). Values shown represent mean ± SE. One-way ANOVA with Bonferroni's multiple comparison test compared to "before radiation" (0 min, ***p < 1.0e–15; 60 min, ***p < 1.0e–15; 360 min, ***p < 1.0e–15).

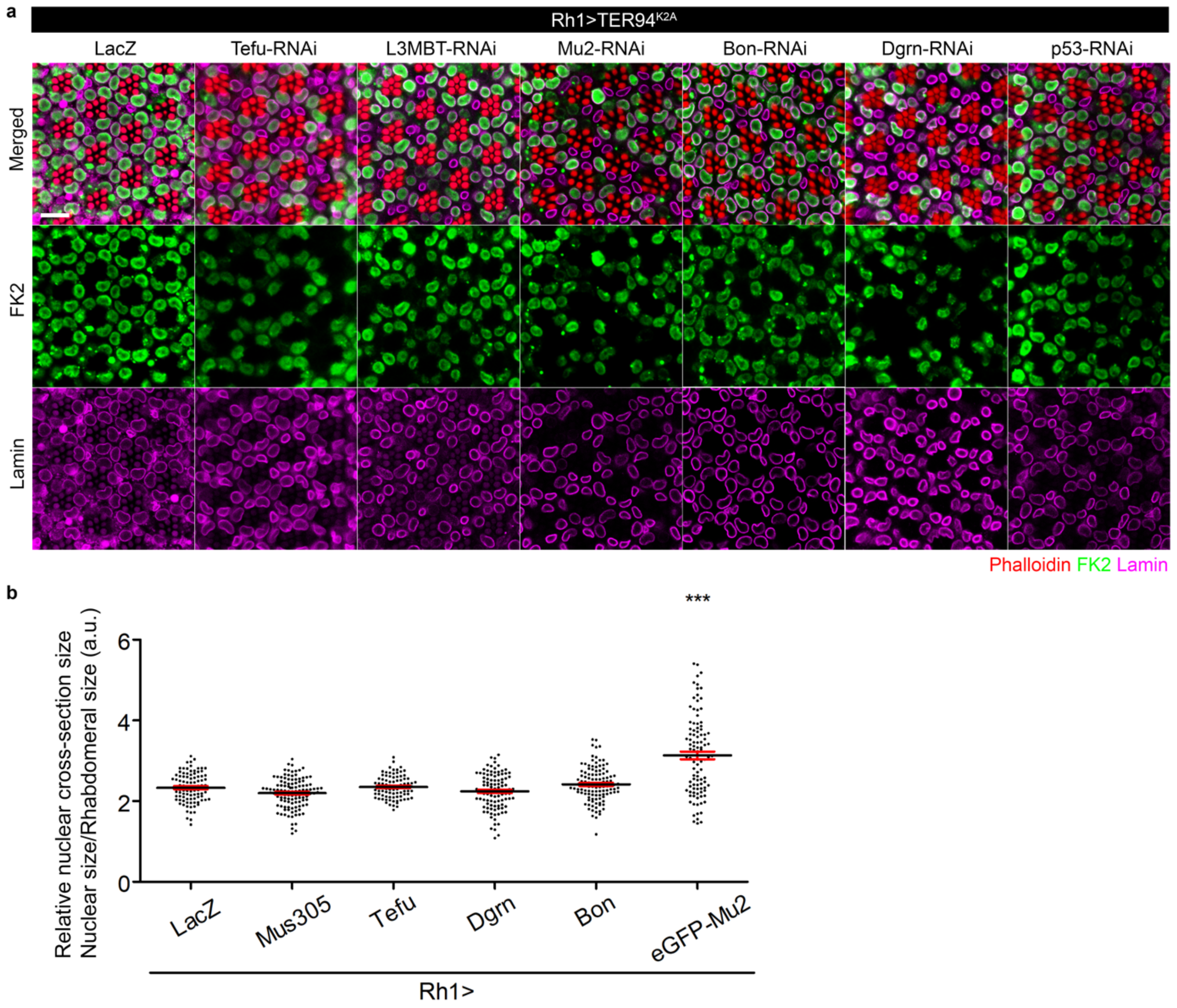

Supplementary Figure 9. A genetic screen targeting DDR genes for ability to modify TER94 dysfunction-induced nuclear expansion. a Confocal images of 5-day-old adult retinas co-expressing TER94^{K2A} with LacZ (control) or the indicated RNAi constructs by *Rh1-GAL4* stained with phalloidin (red), anti-Lamin (magenta), and anti-ubiquitin-conjugates (FK2, green) antibodies. **b** Quantification of the relative nuclear cross-section size of R1-R6 from flies expressing the indicated genes by *Rh1-GAL4*. The number of independent nuclei measured are 93 (LacZ), 117 (Mus305), 90 (Tefu), 103 (Dgrn), 115 (Bon), and 103 (eGFP-Mu2). Values represent mean ± SE. One-way ANOVA with Bonferroni's multiple comparison test compared to LacZ. $p = 0.3081$, ns (Mus305); $p > 0.9999$, ns (Tefu); $p > 0.9999$, ns (Dgrn); $p > 0.9999$, ns (Bon); ***$p < 1.0e{-15}$ (eGFP-Mu2). Scale bar: 10 μm (**a**).

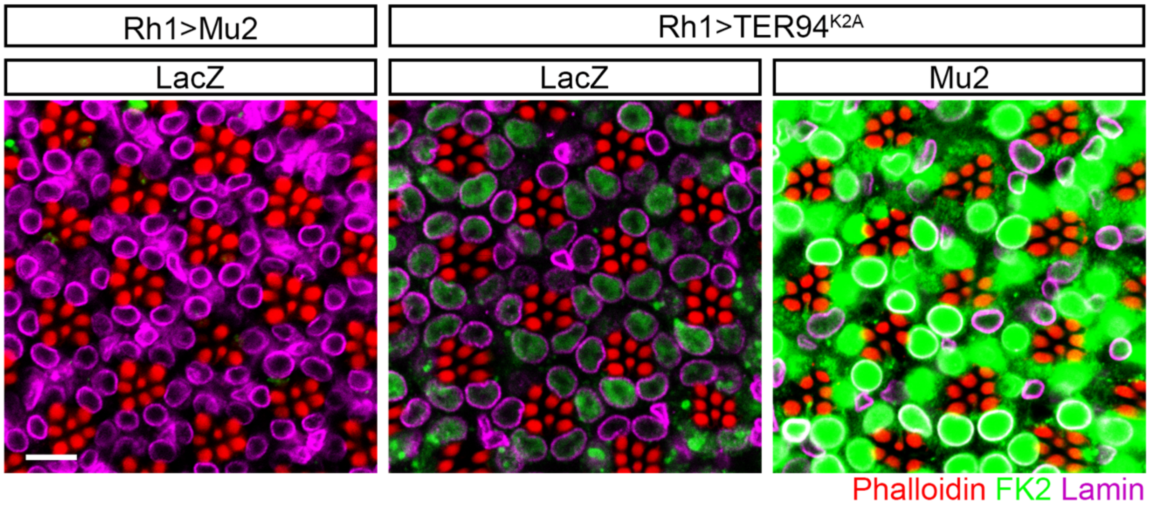

Supplementary Figure 10. Overexpressing Mu2 in TER94 dysfunctional condition escalates nuclear ubiquitinated proteins accumulation. Confocal micrographs of 5-day-old *Rh1>Mu2, LacZ, Rh1>TER94*^{K2A}*, LacZ, and Rh1>TER94*^{K2A}*, Mu2* adult retina stained with phalloidin (red), anti-Lamin (magenta), and anti-ubiquitin-conjugates (FK2, green) antibodies. Images are representative of two independent experiments. Scale bar: 10 µm.

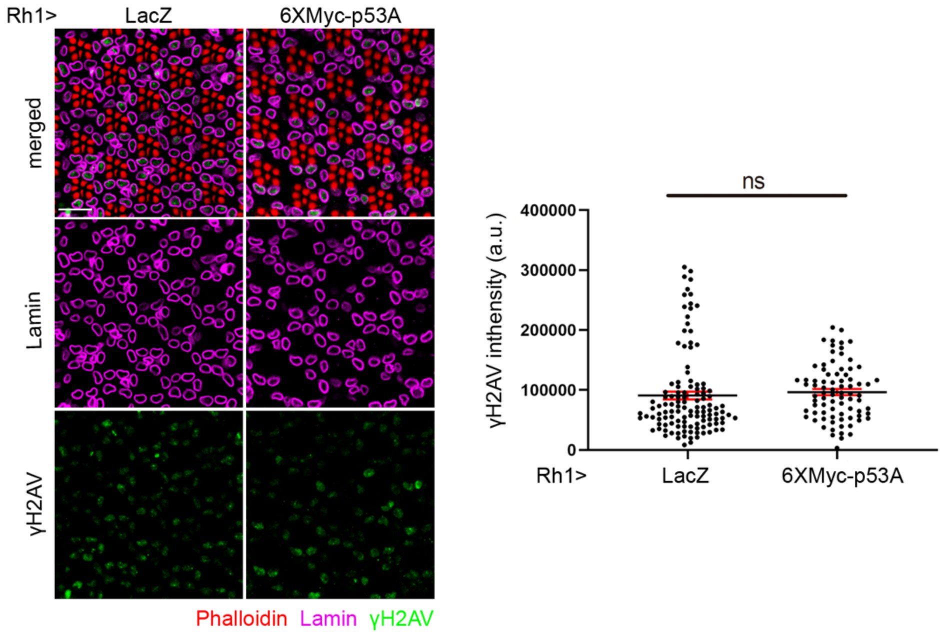

Supplementary Figure 11. Ectopic expression of p53A does not elevate DSB. Confocal micrographs of 5-day-old *Rh1>LacZ and Rh1>6Myc-p53A* adult retina stained with phalloidin (red), anti-Lamin (magenta), and anti-γH2AV (green) antibodies. Flies are in endogenous *p53* heterozygous background. Scale bar: 10 μm. Quantification of γH2AV signal intensity from the indicated genotypes. The number of independent nuclei measured are 164 (LacZ) and 148 (*6Myc-p53A*). Values represent mean ± SE (Student's t-test, two-tailed, p = 0.2778, ns, not significant).

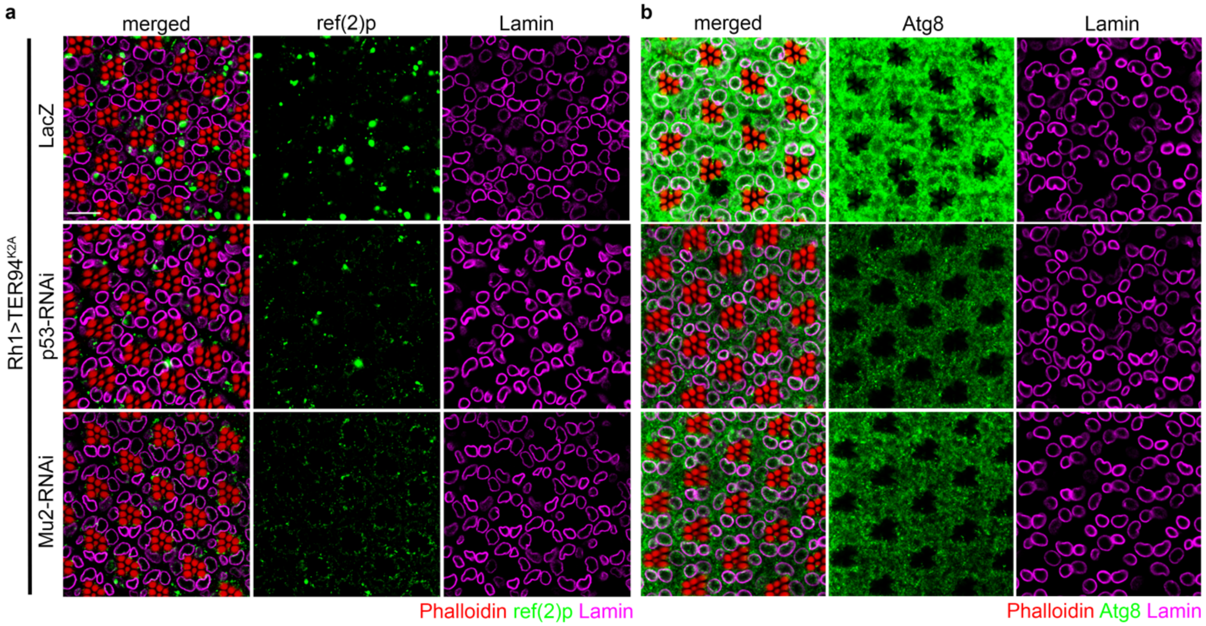

Supplementary Figure 12. Autophagic markers accumulation caused by TER94 dysfunction can be mitigated by Mu2 or p53 knockdown. a, **b** Confocal micrographs of 5-day-old *Rh1>TER94^{K2A}* adult retina co-expressing LacZ, p53-RNAi, or Mu2-RNAi stained with phalloidin (red), anti-Lamin (magenta) and either (**a**) anti-ref(2)p (green) or (**b)** anti-Atg8 (green). Images are representative of two independent experiments. Scale bar: 10 μm.

Supplementary Table 1. Primer sets used in RT-PCR.

Targeting cDNA	Sequences of primers
rp49	F: 5'-CCAGTCGGATCGATATGCTAA-3'
	R: 5'-ACGTTGTGCACCAGGAACTT-3'
TER94	F: 5'-GTGTTCATCATCGGAGCCAC-3'
	R: 5'-GGATCGTCCTCGTCCATGTC-3'
Mu2	F: 5'-GCACGTGGTGGAGATCAC-3'
	R: 5'-CGTACAGATCCACGCTGATG-3'
