# Supplementary material for: VCP maintains nuclear size by regulating the DNA damage-associated MDC1–p53–autophagy axis in Drosophila
Source: Nat Commun. 2021 Jul 12;12:4258. doi: 10.1038/s41467-021-24556-0 (PMC8275807; doi:10.1038/s41467-021-24556-0)
Supplement: Supplementary file 4 — Description of additional supplementary files [file 41467_2021_24556_MOESM4_ESM.docx]

Description of additional supplementary information

Title: Supplementary Movie 1.

Description: Representative nuclei of Rh1>LacZ control and Rh1>TER94K2A reconstructed from Z-stacks of anti-Lamin sections.
