# Supplementary material for: VCP maintains nuclear size by regulating the DNA damage-associated MDC1–p53–autophagy axis in Drosophila
Source: Nat Commun. 2021 Jul 12;12:4258. doi: 10.1038/s41467-021-24556-0 (PMC8275807; doi:10.1038/s41467-021-24556-0)
Supplement: Supplementary file 5 — Reporting Summary [file 41467_2021_24556_MOESM5_ESM.pdf]

nature research

Corresponding author(s): Tzu-Kang Sang

Last updated by author(s): Jun 6, 2021

Reporting Summary

Nature Research wishes to improve the reproducibility of the work that we publish. This form provides structure for consistency and transparency in reporting. For further information on Nature Research policies, see our Editorial Policies and the Editorial Policy Checklist.

Statistics

For all statistical analyses, confirm that the following items are present in the figure legend, table legend, main text, or Methods section.

n/a	Confirmed	
☐	☒	The exact sample size (*n*) for each experimental group/condition, given as a discrete number and unit of measurement
☐	☒	A statement on whether measurements were taken from distinct samples or whether the same sample was measured repeatedly
☐	☒	The statistical test(s) used AND whether they are one- or two-sided<br>*Only common tests should be described solely by name; describe more complex techniques in the Methods section.*
☐	☒	A description of all covariates tested
☐	☒	A description of any assumptions or corrections, such as tests of normality and adjustment for multiple comparisons
☐	☒	A full description of the statistical parameters including central tendency (e.g. means) or other basic estimates (e.g. regression coefficient) AND variation (e.g. standard deviation) or associated estimates of uncertainty (e.g. confidence intervals)
☐	☒	For null hypothesis testing, the test statistic (e.g. *F*, *t*, *r*) with confidence intervals, effect sizes, degrees of freedom and *P* value noted<br>*Give P values as exact values whenever suitable.*
☒	☐	For Bayesian analysis, information on the choice of priors and Markov chain Monte Carlo settings
☒	☐	For hierarchical and complex designs, identification of the appropriate level for tests and full reporting of outcomes
☐	☒	Estimates of effect sizes (e.g. Cohen's *d*, Pearson's *r*), indicating how they were calculated

Our web collection on statistics for biologists contains articles on many of the points above.

Software and code

Policy information about availability of computer code

Data collection	ZEISS ZEN confocal microscopy imaging system was used to collect all the fluorescent images.
Data analysis	Fiji, an image processing package based on Image J 1.53e, was used to measure the sizes of the rhabdomeres, the nuclei, and the fluorescence intensities within the region of interests. Avizo 9.4 (Thermo Fisher Scientific) was used for nucleus volumetric segmentation and volume/morphology quantification. GraphPad Prism 9 was used to analyze the statistical significance of all the data in this study. Adobe Photoshop 2021 was used to prepare figures.

For manuscripts utilizing custom algorithms or software that are central to the research but not yet described in published literature, software must be made available to editors and reviewers. We strongly encourage code deposition in a community repository (e.g. GitHub). See the Nature Research guidelines for submitting code & software for further information.

Data

Policy information about availability of data

All manuscripts must include a data availability statement. This statement should provide the following information, where applicable:

- Accession codes, unique identifiers, or web links for publicly available datasets
- A list of figures that have associated raw data
- A description of any restrictions on data availability

All data support the findings of this study are included in this published article and its Supplementary information files. The raw data underlying Figs. 1c-d, 1f, 2b, 3b, 3e, 3h-i, 4a, 4c-e, 4g-k, 5a-e, 5g, 6c, 6e-k and Supplementary Figs 1b, 2b, 2d, 6b-c, 8b, 8d, 9b, 11 are available in the Source Data file.

Field-specific reporting

Please select the one below that is the best fit for your research. If you are not sure, read the appropriate sections before making your selection.

☒ Life sciences ☐ Behavioural & social sciences ☐ Ecological, evolutionary & environmental sciences

For a reference copy of the document with all sections, see nature.com/documents/nr-reporting-summary-flat.pdf

Life sciences study design

All studies must disclose on these points even when the disclosure is negative.

Sample size	No statistical method was used to predetermine the sample size. Samples sizes were selected based on previous experience to obtain statistical significance and reproducibility. (Chang et al. PLoS Genet. 2011 Feb 3;7(2):e1001288.; Liang et al. PLoS Genet. 2014 Sep 25;10(9):e1004675.)
Data exclusions	No data were excluded from the analyses.
Replication	The experimental findings were successfully reproduced as indicated by the control groups from at least two independent experiments throughout the manuscript.
Randomization	Experimental groups are allocated according to the genetic backgrounds of the samples. Samples observed were of the same age among each group.
Blinding	For all experiments required manually measurement and statistical analysis, including the measurement of rhabdomeres, nuclei sizes or volumes, or the fluorescence intensities in the regions of interest, naive examiners were blinded to the genotypes. For immunohistochemistry, RT-PCR, and western blotting, all the samples were treated with the same procedure and thus blinding is not relevant.

Reporting for specific materials, systems and methods

We require information from authors about some types of materials, experimental systems and methods used in many studies. Here, indicate whether each material, system or method listed is relevant to your study. If you are not sure if a list item applies to your research, read the appropriate section before selecting a response.

Materials & experimental systems

n/a	Involved in the study	
☐	☒	Antibodies
☒	☐	Eukaryotic cell lines
☒	☐	Palaeontology and archaeology
☐	☒	Animals and other organisms
☒	☐	Human research participants
☒	☐	Clinical data
☒	☐	Dual use research of concern

Methods

n/a	Involved in the study	
☒	☐	ChIP-seq
☒	☐	Flow cytometry
☒	☐	MRI-based neuroimaging

Antibodies

Antibodies used	To generate polyclonal anti-TER94, anti-p53, and anti-Mu2 antibodies, polypeptides corresponding to the amino acids 701-801 of TER94, amino acids 373-495 of p53, and the amino acids 627-947 of Mu2 respectively were bacterially expressed, purified, and used for immunization (GeneTex). Mouse anti-Lamin Dm0 (Developmental Studies Hybridoma Bank, DSHB, cat# ADL67.10), rabbit anti-Lamin Dm0 (a gift from Dr. Paul Fisher), mouse anti-Nuclear Pore Complex Proteins (NPC) (Abcam, cat# ab24609, clone# Mab414), mouse anti- mono- and polyubiquitinated conjugates (Enzo Life Sciences, cat# BML-PW8810-0100, clone# FK2), rabbit anti-ref(2)p (Abcam, cat# ab178440), rabbit anti-LC3A/B (Abcam, cat# ab128025), rabbit anti-V5 (Millipore, cat# AB3792), rabbit anti-Atg8 (Millipore, cat#ABC974), mouse anti-γH2Av (DSHB, cat# UNC93-5.2.1), mouse anti-PCNA (Abcam, cat# ab29, clone# PC10), mouse anti-GFP (DSHB, cat# 4C9), mouse anti-Myc (GeneTex, cat# GTX75953, clone# 9E10), rabbit anti-mCherry (GeneTex, cat# GTX128508), rabbit anti-cleaved PARP (Abcam, cat# ab2317), rabbit anti-cleaved Caspase 3 (Cell Signaling, cat# 9661), Phalloidin-Tetramethylrhodamine B isothiocyanate (Sigma, cat# P1951), rabbit anti-Histone H3 (Abcam, cat# ab1791), mouse anti-β-actin (GeneTex, cat# GTX629630, Clone# GT5512), rabbit anti-GFP (GeneTex, cat# GTX113617), mouse anti-Myc (GeneTex, cat#GTX75953), Goat anti-Rabbit IgG (HRP) (GeneTex, cat# GTX213110-01), Goat anti-Mouse IgG (HRP) (GeneTex, cat# GTX213111-01), Alexa Fluor® 488 AffiniPure Goat Anti-Mouse IgG (H+L) (Jackson ImmunoResearch Laboratories, cat#115-545-146), Alexa Fluor® 488 AffiniPure Goat Anti-Rabbit IgG (H+L) (Jackson ImmunoResearch Laboratories, cat#111-545-144), Alexa Fluor® 647 AffiniPure Goat Anti-Mouse IgG (H+L) (Jackson ImmunoResearch Laboratories, cat#115-605-146), Cy™3 AffiniPure Goat Anti-Mouse IgG (H+L) (Jackson ImmunoResearch Laboratories, cat#115-165-146), Cy™5 AffiniPure Goat Anti-Mouse IgG (H+L) (Jackson ImmunoResearch Laboratories, cat#115-175-146), Cy™3 AffiniPure Goat Anti-Rabbit IgG (H+L) (Jackson ImmunoResearch Laboratories, cat#111-165-144), Cy™5 AffiniPure Goat Anti-Rabbit IgG (H+L) (Jackson ImmunoResearch Laboratories, cat#111-175-144).

Validation	The validation of anti-TER94 is described in Chang et al., PLoS Genet (2011). The validation of anti-p53 and anti-Mu2 for recognizing Drosophila p53 and Mu2, respectively in western blotting was confirmed by samples with or without the overexpression of Drosophila p53 or Mu2. The validation of mouse anti-Nuclear Pore Complex Proteins(NPC) for recognizing Drosophila NPC was confirmed by the co-localization of anti-NPC signal with anti-Lamin as Fig.1e showed. The validation of rabbit anti-Lamin Dm0 is described in Furukawa, K. et al., J Cell Sci (2003). The validation of rabbit anti-cleaved Caspase 3 is described in Liang et al. PLoS Genet (2014). All the other antibody validation of immunoblotting or immunofluorescence are provided on the manufacturer's websites: mouse anti-γH2AV (Drosophila), mouse anti-GFP (Aequorea victoria, all species for fusion proteins), rabbit anti-ref(2)p (Drosophila melanogaster), rabbit anti LC3A/B (Reacts with: Mouse, Human. Predicted to work with: Rat, Cow, Drosophila melanogaster, Nonhuman primates), mouse anti-PCNA(Reacts with: Mouse, Rat, Human. Predicted to work with: Chicken, Cow, Pigeon, Pig, Drosophila melanogaster, Monkey, Zebrafish, Thornback ray, Dogfish, Catshark), rabbit anti-cleaved PARP (Human), rabbit anti-Histone H3 (Reacts with: Mouse, Rat, Human, Saccharomyces cerevisiae, Xenopus laevis, Arabidopsis thaliana, Drosophila melanogaster, Indian muntjac, Schizosaccharomyces pombe. Predicted to work with: Chicken, Dog, Caenorhabditis elegans, Ferret, Zebrafish, a wide range of other species, Mammals, Silk worm, Dictyostelium discoideum, Rainbow trout, Neurospora crassa, Toxoplasma gondii, Rice, Schistosoma mansoni, Candida albicans, Cyanidioschyzon merolae), mouse anti-β-action (Human, Mouse, Rat, Zebrafish, Yeast, Rabbit, Goat, Drosophila, Dog, Xenopus), rabbit anti-atg8 (Drosophila), mouse anti-mono-and polyubiquitinated conjugates (Species independent). rabbit anti-GFP, rabbit anti-mCherry, rabbit anti-V5, mouse anti-Myc recognize all species for fusion proteins.

Animals and other organisms

Policy information about studies involving animals; ARRIVE guidelines recommended for reporting animal research

Laboratory animals	Drosophila melanogaster third instar larva, pupa, and adults aged from freshly eclosed to 8-day-old, both males and females, were used in this study. Drosophila strains used were: Caton-S (CS), UAS-LacZ, Rh4-GAL4, Spa-GAL4, Mhc-GAL4, UAS-tub-GAL80ts, UAS-GFP-NLS, hs-FLP, FRT42D GMR-myr.GFP, UAS-CD4-mCherry, UAS-EGFP.Mu2, Fly-FUCCI (UAS-GFP-E2F11-230, UAS-mRFP1-NLS-CycB1-266), FRT42D TER94K15502, UAS-S/G2/M-Green, GMR-GAL4, Rh1-GAL4, UAS-CD8-PARP-Venus, UAS-GFP-Atg8a and UAS-GFP-p62, UAS-6XMyc-p53 isoforms, UAS-CD3δ-YFP, UAS-TER94WT, UAS-TER94A229E, UAS-TER94K2A, UAS-TER94E2Q, UAS-Derlin-1, UAS-Derlin-1ΔSHP, UAS-Mu2, p53 5A-1-4, UAS-GFP-mCherry-Atg8a, UAS-Bon-RNAi, UAS-Blm-RNAi, UAS-CG12728 (NHEJ1)-RNAi, UAS-CG2990-RNAi, UAS-CG3448 (XRCC4)-RNAi, UAS-CG32756-RNAi, UAS-CG5825-RNAi, UAS-CG5872-RNAi, UAS-CG9272-RNAi, UAS-dgrn-RNAi, UAS-ERCC1-RNAi, UAS-GEN-RNAi, UAS-HDM-RNAi, UAS-Ku80-RNAi, UAS-Irbp-RNAi, UAS-L3MBT-RNAi, UAS-Mu2-RNAi, UAS-mms4-RNAi, UAS-Mei-9-RNAi, UAS-Mre11-RNAi, UAS-MBD-R2-RNAi, UAS-Mei-41-RNAi, UAS-mus81-RNAi, UAS-Mus101-RNAi, UAS-Mus205-RNAi, UAS-Mus301-RNAi, UAS-Mus304-RNAi, UAS-Mus312-RNAi, UAS-Mus308-RNAi, UAS-CG9272-RNAi, UAS-NBS-RNAi, UAS-Ogg1-RNAi, UAS-Okr-RNAi, UAS-p53-RNAi, UAS-Rad50-RNAi, UAS-Rad51D-RNAi, UAS-RPS3-RNAi, UAS-Spn-A-RNAi, UAS-Spn-B-RNAi, UAS-Spn-D-RNAi, UAS-Swm1-RNAi, UAS-Slx1-RNAi, UAS-Tos-RNAi, UAS-TOP3-RNAi, UAS-Thd1-RNAi, UAS-Xpc-RNAi, UAS-Tefu-RNAi, UAS-XRCC2-RNAi, and UAS-Lig4-RNAi.
Wild animals	This study did not involve wild animals.
Field-collected samples	This study did not involve samples collected from the field.
Ethics oversight	This study only involved Drosophila melanogaster, which does not require ethical approval.

Note that full information on the approval of the study protocol must also be provided in the manuscript.
